# Comparison of patients hospitalized with COVID-19, H7N9 and H1N1

**DOI:** 10.1186/s40249-020-00781-5

**Published:** 2020-12-02

**Authors:** Li-Si Deng, Jing Yuan, Li Ding, Yuan-Li Chen, Chao-Hui Zhao, Gong-Qi Chen, Xing-Hua Li, Xiao-He Li, Wen-Tao Luo, Jian-Feng Lan, Guo-Yu Tan, Sheng-Hong Tang, Jin-Yu Xia, Xi Liu

**Affiliations:** 1 Department of Infectious Diseases, The Fifth Affiliated Hospital, Sun Yat-Sen University, Zhuhai, 519000 China; 2 Diagnosis and Treatment of Infectious Diseases Research Laboratory, Shenzhen Third People’s Hospital, Shenzhen, 518112 China; 3 Department of Hospital Infection Control, The Fifth Affiliated Hospital, Sun Yat-Sen University, Zhuhai, 519000 China

**Keywords:** SARS-CoV-2, COVID-19, H7N9, H1N1, Comparison

## Abstract

**Background:**

There is an urgent need to better understand the novel coronavirus, severe acute respiratory syndrome coronavirus 2 (SARS-CoV-2), for that the coronavirus disease 2019 (COVID-19) continues to cause considerable morbidity and mortality worldwide. This paper was to differentiate COVID-19 from other respiratory infectious diseases such as avian-origin influenza A (H7N9) and influenza A (H1N1) virus infections.

**Methods:**

We included patients who had been hospitalized with laboratory-confirmed infection by SARS-CoV-2 (*n* = 83), H7N9 (*n* = 36), H1N1 (*n* = 44) viruses. Clinical presentation, chest CT features, and progression of patients were compared. We used the Logistic regression model to explore the possible risk factors.

**Results:**

Both COVID-19 and H7N9 patients had a longer duration of hospitalization than H1N1 patients (*P* < 0.01), a higher complication rate, and more severe cases than H1N1 patients. H7N9 patients had higher hospitalization-fatality ratio than COVID-19 patients (*P* = 0.01). H7N9 patients had similar patterns of lymphopenia, neutrophilia, elevated alanine aminotransferase, C-reactive protein, lactate dehydrogenase, and those seen in H1N1 patients, which were all significantly different from patients with COVID-19 (*P* < 0.01). Either H7N9 or H1N1 patients had more obvious symptoms, like fever, fatigue, yellow sputum, and myalgia than COVID-19 patients (*P* < 0.01). The mean duration of viral shedding was 9.5 days for SARS-CoV-2 vs 9.9 days for H7N9 (*P* = 0.78). For severe cases, the meantime from illness onset to severity was 8.0 days for COVID-19 vs 5.2 days for H7N9 (*P* < 0.01), the comorbidity of chronic heart disease was more common in the COVID-19 patients than H7N9 (*P* = 0.02). Multivariate analysis showed that chronic heart disease was a possible risk factor (*OR* > 1) for COVID-19, compared with H1N1 and H7N9.

**Conclusions:**

The proportion of severe cases were higher for H7N9 and SARS-CoV-2 infections, compared with H1N1. The meantime from illness onset to severity was shorter for H7N9. Chronic heart disease was a possible risk factor for COVID-19.The comparison may provide the rationale for strategies of isolation and treatment of infected patients in the future.

## Background

The emergence of human infections with the SARS-CoV-2 (Severe Acute Respiratory Syndrome Coronavirus 2) virus and its rapid national and international spread poses a global health emergency [1]. As of 15 April 2020, the number of patients infected with SARS-CoV-2 has exceeded two million globally, with the highest mortality rate of beyond 10.0% in several countries. Although the outbreak was likely to have started from a zoonotic transmission and associated with live wild animals, it has soon been confirmed that direct human-to-human transmission was occurring [2].

It has been reported that highest viral loads (inversely related to CT value) were detected soon after symptom onset, with higher viral loads detected in the nose than in the throat; it has suggested that the viral nucleic acid shedding pattern of patients infected with SARS-CoV-2 resembles that of patients with influenza and appears different from that seen in patients infected with SARS-CoV [3]. Besides, the pattern of transmission and the characteristics of the disease are similar to influenza initially, although they are from different viral families [4]. It may confuse in identifying influenza and COVID-19 (the Coronavirus disease 2019) for that common symptoms include fever and cough, whereas gastrointestinal symptoms (eg, nausea, vomiting, diarrhea) [4]. In the past decade, two highly pathogenic influenza virus, the avian influenza A (H7N9) and influenza A/H1N1/2009 virus have emerged in two separate events.

The pandemic caused by the influenza A/H1N1/2009 virus starting in the spring of 2009 has caused significant morbidity and mortality in certain patients [5, 6]. During the spring of 2013, cases of human infection with avian influenza A (H7N9) virus were first reported in China, evidence emerged in many cities and regions; most of the cases were severe, with high fatality; as of May 9 2013, the World Health Organization (WHO) had reported 131 laboratory-confirmed cases, including 32 deaths [7–9].

Understanding the clinical characteristics and determinants of the severity of disease due to SARS-CoV-2 virus infection is essential both for the identification and clinical management of high-risk cases. To provide insights into the pathogenesis of SARS-CoV-2 virus infection, we compared the clinical presentation, chest CT features, and progression of patients hospitalized with SARS-CoV-2, H7N9, and H1N1 virus infections. We also compared the characteristics of severe cases between COVID-19 and H7N9, severe cases with H1N1 were not included in the comparison because the number was just five and small amounts of data may not be representative.

## Methods

### Study design and participants

The patients with laboratory confirmed SARS-CoV-2 infection were hospitalized between 17 January 2020 and 20 March 2020, and H1N1 virus infections were hospitalized between 20 March 2017 and 8 March 2019, at The Fifth Affiliated Hospital of Sun Yat-Sen University. Patients with H7N9 virus infection were hospitalized between 18 December 2013 and 28 Febuary 2015, at Shenzhen Third People's Hospital. All subjects with virus infection reported in this manuscript were hospitalized patients and had been laboratory confirmed by real-time reverse transcriptase polymerase chain reaction (RT-PCR). Besides, all patients with H1N1and H7N9 had been hospitalized for pneumonia or severe symptoms (eg, uncontrollable fever, shortness of breath, severe cough, hemoptysis, symptoms associated with comorbidities); but for hospitalized COVID-19 cases, only patients with pneumonia were included in the analysis.

### Data collection

All the clinical data on signs and symptoms, underlying comorbidities, laboratory results, chest CT scans, and treatment measures were retrospectively extracted from electronic medical records and checked by both on-site and off-site doctors. We extracted the baseline data from the patients after admission. The RT-PCR test was performed using nasal and pharyngeal swab specimens, RT-PCR was performed every other day and three consecutive days once negative for SARS-CoV-2 test after admission to hospital, RT-PCR was performed every other day and two consecutive days once negative for H7N9′s test. Chest CT scans were performed on admission (except for two pregnant patients with H1N1 virus infections), and analyzed according to the number of lung lobes involvement. The total CT score was the sum of the individual lobar involvement. Fever was defined as the axillary temperature of at least 37.3 °C. Severe cases were defined by the patient experiencing an oxygenation index under 300 mmHg or admission to an intensive care unit.

### Statistical analysis

We used *χ*^2^ and Fischer’s exact tests for categorical variables, whereas we used the student’s *t*-test or Mann–Whitney *U* test for continuous variables to assess the differences. We did statistical analyses using SPSS software (version 13.0 SPSS, Chicago, Illinois). The significance for all statistical analyses was defined as *P* < 0.05. We used the Kaplan–Meier method to estimate survival curves for death. The same approach was used to determine the time for invasive mechanical ventilation or tracheal intubation. The Logistic regression model was used to explore the possible risk factors. We used kernel density to determine the distribution of the number of days of hospitalization, and the days from illness onset to severity. Severity was defined by the patient experiencing an oxygenation index under 300 mmHg or or admission to an intensive care unit.

## Results

### Patient characteristics

**Fig. 1:**
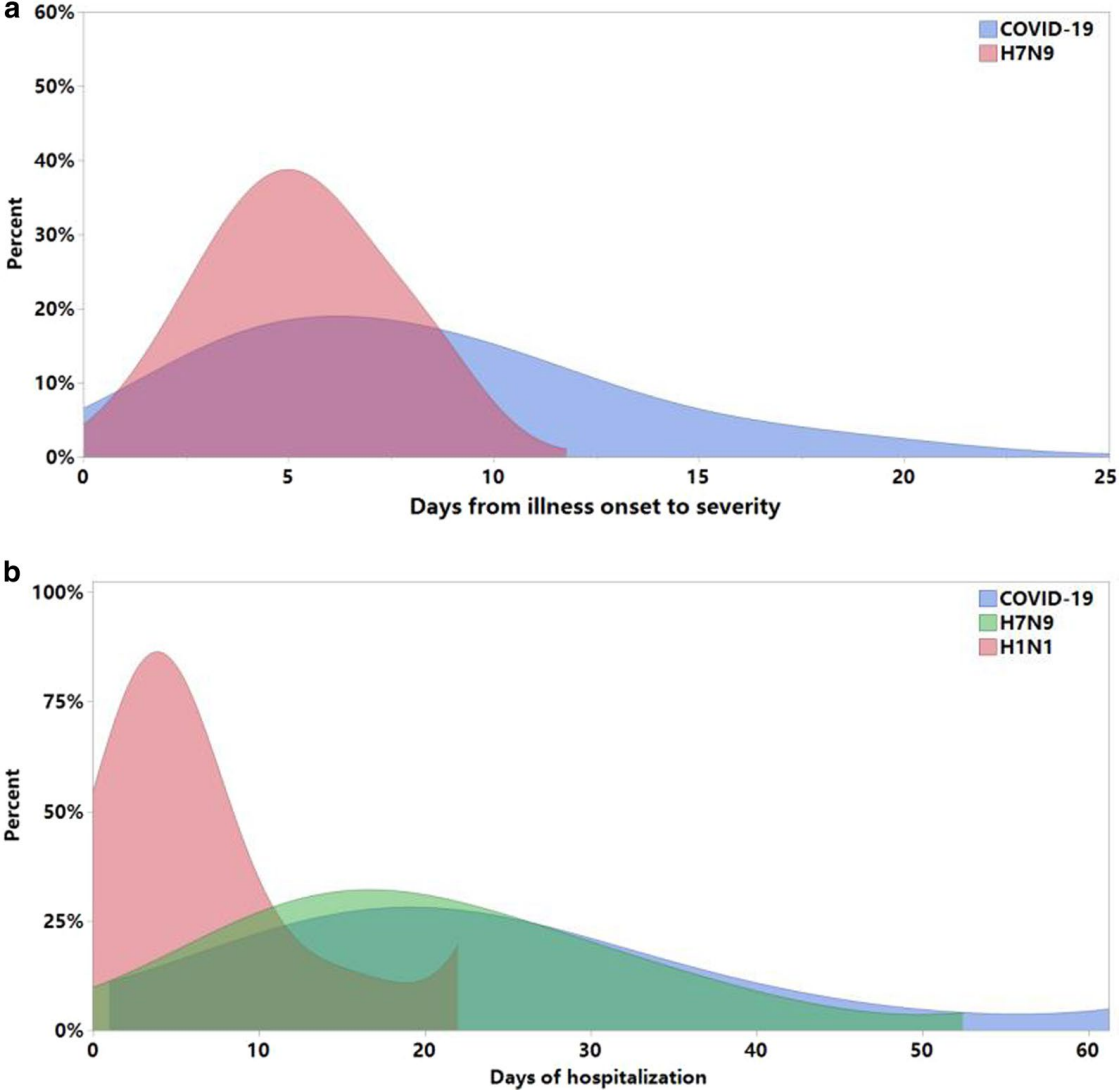
**a** Distribution of the number of days of hospitalization for patients with COVID-19, H7N9 and H1N1. **b** The days from illness onset to severity for severe patients with COVID-19 and H7N9. COVID-19: Coronavirus disease 2019, H7N9: avian-origin influenza A (H7N9) virus, H1N1: influenza A (H1N1) virus

Data were included 83 patients with COVID-19, 36 patients with H7N9, and 44 patients with H1N1 virus infections. The median age of subjects hospitalized with COVID-19 was 46.5 years, compared to 54.5 years for H7N9 patients and 48.0 years for H1N1 patients (Table 1). The prevalence of diabetes was lower in subjects with COVID-19 compared with the H7N9 group. Subjects hospitalized with H7N9 and H1N1 had the highest prevalence of hypertension and smoking. But, no other statistically significant differences in chronic medical conditions were noted between the three groups. Before we collect the data, all the patients hospitalized with COVID-19 were discharged from hospital except for one death. Both COVID-19 and H7N9 patients had a longer duration of hospitalization than H1N1 patients (*P* < 0.01) (Fig. 1), a higher complication rate, and more severe cases than H1N1 patients. H7N9 patients had higher hospitalization-fatality ratio than COVID-19 patients (*P* = 0.01) (Table 1).

**Table 1:** Characteristics of Patients hospitalized with COVID-19, H7N9, and H1N1

Variable	COVID-19 (*n* = 83)	H7N9 (*n* = 36)	*P* value*	H1N1 (*N* = 44)	*P* value^#^
*Characteristic*					
Age, years, median (range)	53 (3–80)	54.5 (21–82)	0.24	48.0 (16–84)	0.69
Male	35 (42.2%)	23 (63.9%)	0.04	26 (59.1%)	0.12
Chronic heart disease	22 (26.5%)	5 (13.8%)	0.16	4 (9.1%)	0.02
Chronic lung disease	3 (3.6%)	1 (2.8%)	1	2 (4.5%)	1
Chronic renal disease	3 (3.6%)	1 (2.8%)	1	7 (15.9%)	0.03
Chronic liver disease	3 (3.6%)	3 (8.3%)	0.37	3 (6.8%)	0.42
Diabetes	7 (8.4%)	9 (25.0%)	0.02	3 (6.8%)	1
Hypertension	15 (18.1%)	14 (38.9%)	0.02	13 (29.5%)	0.18
Malignancy	4 (4.8%)	0 (0%)	0.31	4 (9.1%)	0.45
Smoking history	3 (3.6%)	6 (16.7%)	0.02	8 (18.2%)	0.02
Hospital stays	20.7 (8.5)	19.5 (9.0)	0.28	5.3 (4.1)	< 0.01
Severe cases	29 (34.9%)	30 (83.3%)	< 0.01	5 (11.4%)	< 0.01
Dead	1 (1.2%)	5 (13.9%)	0.01	1 (2.3%)	1
Glucocorticoids	10 (12.0%)	28 (77.8)	< 0.01	0	0.02
Antiviral	83 (100%)	36 (100%)	1	44 (100%)	1
Antibiotics	53 (63.9%)	36 (100%)	< 0.01	25 (56.8%)	0.45
Complication	27 (32.5%)	31 (86.1%)	< 0.01	6 (13.6%)	0.03
*Symptoms*					
Fever (≥ 37.3 ℃)	60 (72.3%)	36 (100%)	< 0.01	44 (100%)	< 0.01
Any cough	70 (84.3%)	34 (94.4%)	0.15	40 (91%)	0.41
Dry cough	64 (77.1%)	30 (83.3%)	0.44	25 (56.8%)	0.02
Yellow sputum	6 (7.2%)	19 (52.8%)	< 0.01	15 (34.1%)	< 0.01
Hemoptysis	1 (1.2%)	8 (22.2%)	< 0.01	0 (0%)	1
Myalgia	10 (12.0%)	12 (33.3%)	< 0.01	17 (38.6%)	< 0.01
Fatigue	11 (13.3%)	14 (38.9%)	< 0.01	25 (56.8%)	< 0.01
Shortness of breath	5 (6.0%)	6 (16.7%)	0.07	5 (11.4%)	0.29
Gastrointestinal symptoms	10 (12.0%)	7 (19.4%)	0.39	16 (36.4%)	< 0.01
*Laboratory findings and CT images*					
AST	23.5 (9.6)	100.7 (110.7)	< 0.01	29.4 (13.9)	< 0.01
ALT	22.5 (15.6)	58.7 (55.4)	< 0.01	19.3 (10.9)	0.13
CK	93.4 (107.6)	796.5 (1367.3)	< 0.01	207.8 (336.4)	< 0.01
LDH	187.8 (54.6)	701.9 (484.2)	< 0.01	213.5 (74.8)	0.01
Leukopenia	15 (18.1%)	7 (19.4%)	0.86	3 (6.8%)	0.11
Lymphopenia	25 (30.1%)	32 (88.9%)	< 0.01	23 (52.2%)	0.01
Neutropenia	21 (25.3%)	2 (5.6%)	0.01	3 (6.8%)	0.02
Neutrophilia	2 (2.4%)	9 (25%)	< 0.01	8 (18.2%)	< 0.01
Thrombocytopenia	8 (9.6%)	14 (38.9%)	< 0.01	4 (9.1%)	0.92
Elevated CRP	22 (26.5%)	35 (97.2%)	< 0.01	30 (68.2%)	< 0.01
Duration of viral shedding	9.5 (6.1)	9.9 (6.2)	0.78	NA	NA
Lung lobes involvement	3 (1–5)	5 (1–5)	< 0.01	0 (0–5)	< 0.01

Data are presented as mean (SD), medians (interquartile ranges) or No. (%). *P* value*: compared “COVID-19” and “H7N9”, *P* value^**#**^: compared “COVID-19” and “H1N1”

*ALT* alanine aminotransferase, *AST* aspartate transaminase, *CK* creatine kinase, *LDH* lactate dehydrogenas, *CRP* C-reactive protein

Either H7N9 or H1N1 patients had more obvious symptoms, like fever, fatigue, yellow sputum, and myalgia than COVID-19 patients (*P* < 0.01). Gastrointestinal symptoms were most common in H1N1 cases, while hemoptysis symptoms were most common in H7N9 cases (Table 1). H7N9 patients had similar patterns of lymphopenia, neutrophilia, elevated alanine aminotransferase, C-reactive protein, and those seen in H1N1 patients, which were all significantly different from patients with COVID-19 (*P* < 0.01) (Table 1). Thrombocytopenia was more common in patients with H7N9, elevated lactate dehydrogenase was equally common in H7N9 and H1N1 patients, and least common in COVID-19 patients. But, no other statistically significant differences in baseline characteristics.

### Comorbidity and risk factors

Compared patients hospitalized with COVID-19, the prevalence of hypertension and diabetes were higher in subjects with H7N9 (*P* = 0.02), whereas the prevalence of chronic renal disease was higher in subjects with H1N1 (*P* = 0.03). A history of smoking was more common in subjects hospitalized with H7N9 and H1N1 (Table1). Chronic heart disease more frequent in subjects with COVID-19, compared with H1N1 (Table 1), whereas for severe cases, COVID-19 had the highest prevalence of chronic heart disease than H7N9 (*P* = 0.02) (Table 2). Multivariate analysis showed that chronic heart disease was a possible risk factor (*OR* > 1) for COVID-19, compared with H1N1 and H7N9 (Additional file 1: Table S1).

**Table 2:** Characteristics of severe patients with COVID-19 or H7N9

Variable	COVID-19 (*n* = 29)	H7N9 (*n* = 30)	*P* value
*Characteristic*			
Age, years, median (range)	59 (32–80)	56 (21–82)	0.98
Male	15 (51.7%)	20 (66.7%)	0.29
Chronic heart disease	12 (41.4%)	4 (13.3%)	0.02
Chronic lung disease	2 (6.9%)	1 (3.3%)	0.61
Chronic renal disease	2 (6.9%)	1 (3.3%)	0.61
Chronic liver disease	0	3(10.0%)	0.24
Diabetes	5 (17.2%)	9 (30.0%)	0.36
Hypertension	9 (31.0%)	14 (46.7%)	0.29
Malignancy	0	0	1.00
Smoking history	1 (3.4%)	5 (16.7%)	0.19
Hospital stays	21.3 (6.9)	20.6 (8.9)	0.71
Dead	1 (3.4%)	5 (16.7%)	0.19
The days from illness onset to severity	8.0 (4.6)	5.2 (2.1)	< 0.01
*Symptoms*			
Fever (≥ 37.3 ℃)	26 (89.7%)	27 (90.0%)	1.00
Any cough	15 (51.7%)	28 (93.3%)	< 0.01
Dry cough	10 (34.5%)	10 (33.3%)	1.00
Yellow sputum	5 (17.2%)	18 (60.0%)	< 0.01
Hemoptysis	1 (3.4%)	8 (26.7%)	0.03
Myalgia	5 (17.2%)	11 (36.7%)	0.14
Fatigue	8 (27.6%)	12 (40.0%)	0.41
Gastrointestinal symptoms	4 (13.8%)	7 (23.3%)	0.51
*Laboratory findings and CT images*			
AST	27.5 (11.1)	111.1 (118.4)	< 0.01
ALT	26.2 (17.4)	61.7 (54.0)	< 0.01
CK	167.5 (31.0)	1272.3 (268.8)	< 0.01
LDH	215.6 (50.6)	745.8 (512.9)	< 0.01
Leukopenia	7 (24.1%)	7 (23.3%)	1.00
Lymphopenia	15 (51.7%)	27 (90.0%)	< 0.01
Neutropenia	9 (31.0%)	2 (6.7%)	0.02
Neutrophilia	1 (3.4%)	28 (93.3%)	< 0.01
Thrombocytopenia	4 (13.8%)	10 (33.3%)	0.12
Elevated CRP	19 (65.5%)	29 (96.7%)	< 0.01
Duration of viral shedding	10.6 (6.6)	10.2 (6.4)	0.86
Lung lobes involvement	4	5	0.14

Data are presented as mean (SD), medians (interquartile ranges) or No. (%)

*ALT* alanine aminotransferase, *AST* aspartate transaminase, *CK* creatine kinase, *LDH* lactate dehydrogenas, *CRP* C-reactive protein

### Characteristics of severe cases with COVID-19 or H7N9

**Fig. 2:**
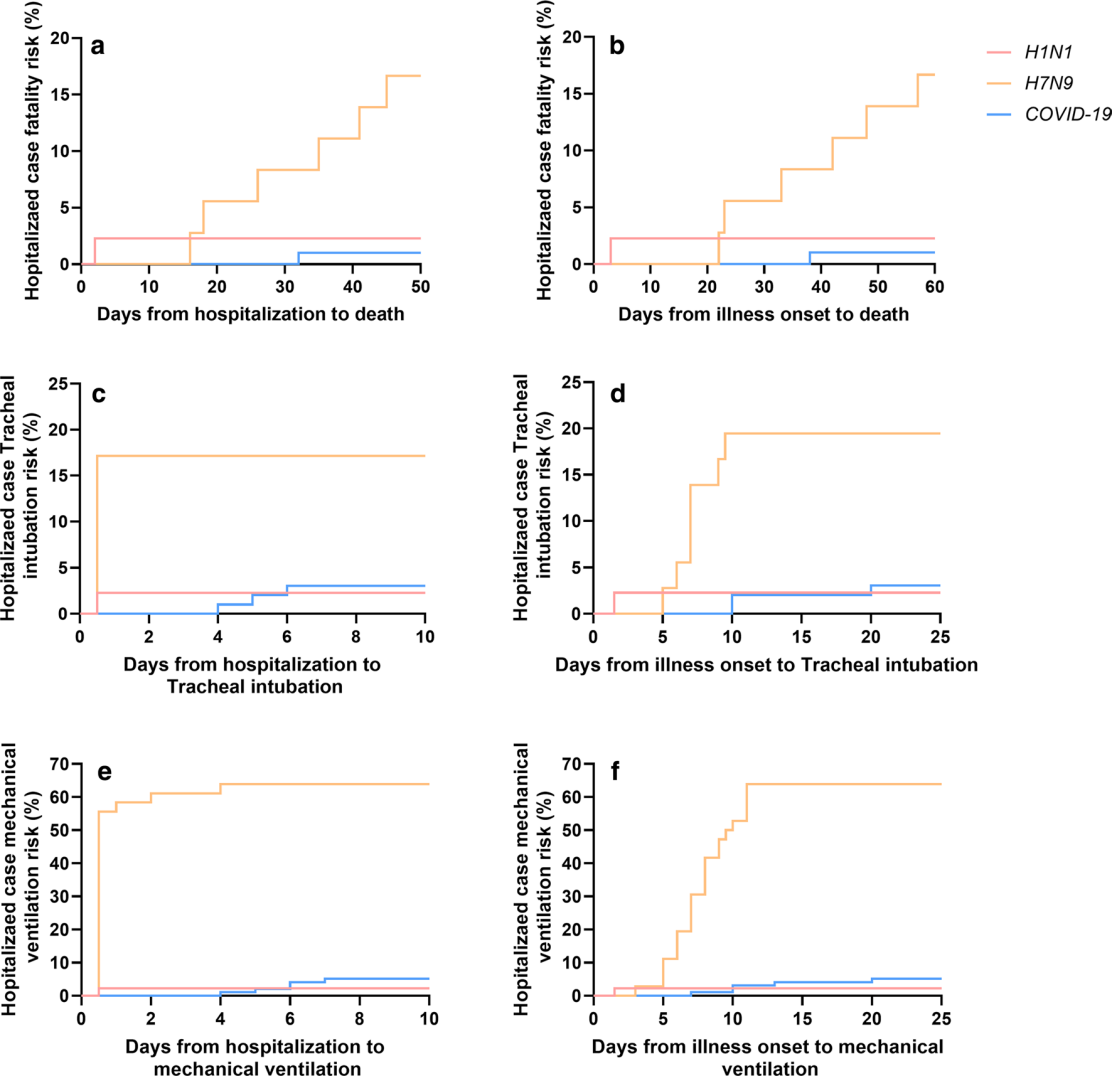
Case fatality risk and invasive ventilation risk in hospitalized patients. **a** Days from hospitalization to death. **b** days from illness onset to death. **c** Days from hospitalization to tracheal intubation. **d** Days from illness onset to tracheal intubation. **e** Days from hospitalization to mechanical ventilation. **f** Days from illness onset to mechanical ventilation

The mean time from illness onset to severity was 8.0 days for COVID-19 vs 5.2 days for H7N9 (*P* < 0.01) (Fig. 1). For the severe cases, there were no significant differences in the hospitalization-fatality ratio and the lung lobes involvement between COVID-19 and H7N9 (Table 2). The days from hospitalization to mechanical ventilation and the days from illness onset to mechanical ventilation in severe cases of COVID-19 patients were more protracted than H7N9 and H1N1 cases (Fig. 2). Symptoms, like cough, yellow sputum and hemoptysis, were more common in severe cases with H7N9 than COVID-19; but, Symptoms, like fever, fatigue myalgia, were equally common in the two groups of severe cases, that was different from the comparison of general cases (Table 2). Except that, the other results of comparison for the severe cases between COVID-19 and H7N9 were similar to the comparison of general cases.

### Chest CT findings

**Fig. 3:**
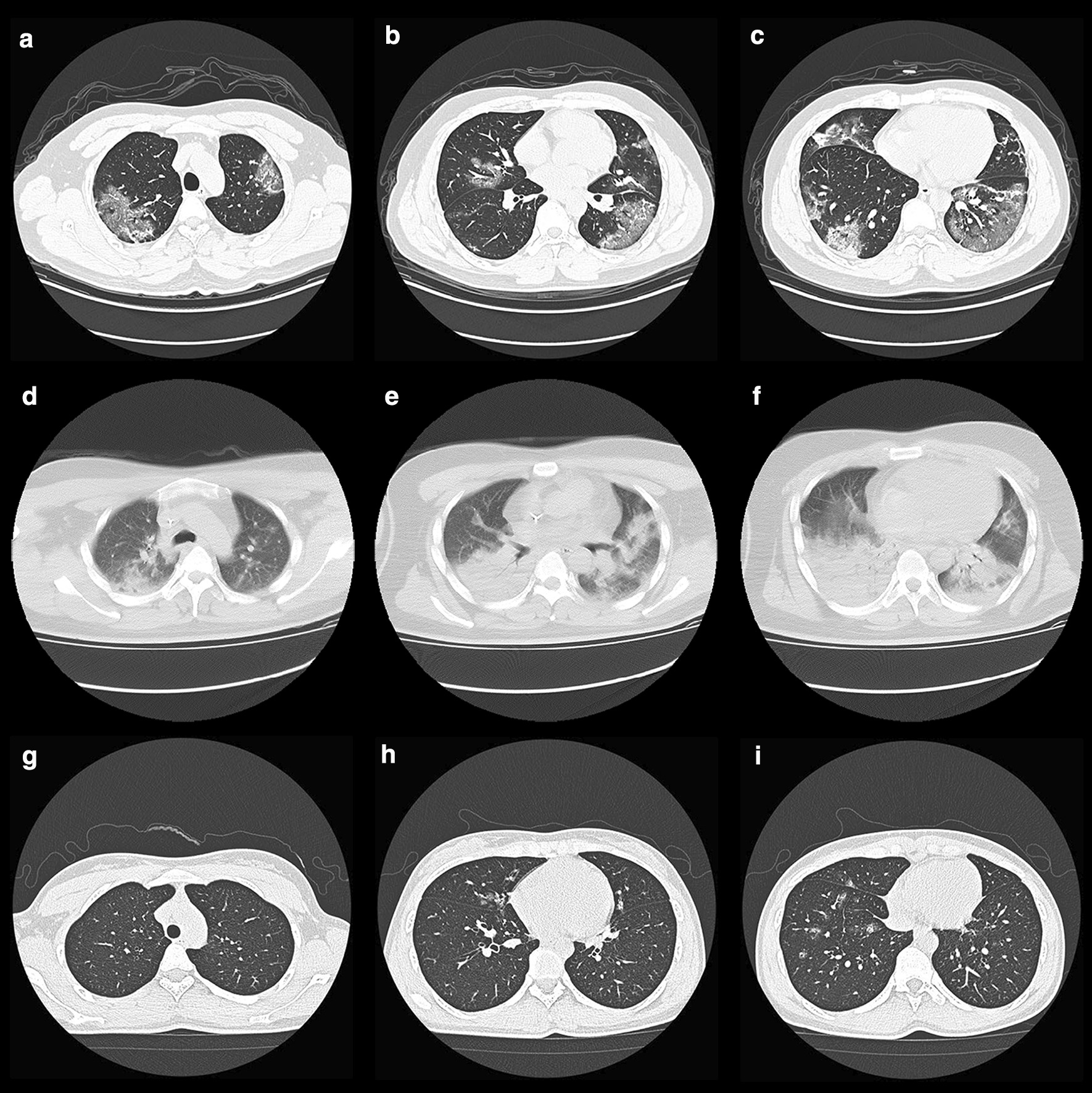
**a**–**c** Chest CT Images of a 36-year-old man with COVID-19 on admission, showed multiple ground-glass densification shadows with multiple diffusions in both lungs, mainly distributed under the pleura. **d**–**f** Chest CT Images of a 32-year-old man infected with H7N9 on admission, showed multilobar patchy consolidation and diffuse alveolar opacities. **g**–**i** Chest CT Images of a 29-year-old woman infected with H1N1 on admission, showed ground-glass opacity and small patchy shadows with diffused distribution in the right middle and lower lungs

The proportion of pneumonia and the number of lobes involved in COVID-19 patients was higher than in H1N1 cases, but lower than in H7N9 cases (Table 1) (Details in Additional file 1: Table S2). In COVID-19 group, 77 (93%) of 83 patients’ chest CT manifestations were multiple ground-glass densification shadows with various diffusions in both lungs, mainly distributed under the pleura indistinct nodules may be presented in some cases (Fig. 3). 81% H7N9 cases showed multilobar uneven consolidation and diffuse alveolar opacities. The chest CT radiological findings in 89% hospitalized H1N1 cases with pneumonia were ground-glass opacity and small patchy shadows with diffused distribution in the right middle and lower lungs.

### Duration of viral shedding

The mean duration of viral shedding for nasopharyngeal specimens was 9.5 days for SARS-CoV-2 vs 9.9 days for H7N9 (*P* = 0.78) (Details in Additional file 1: Table S3); for severe cases, it was 10.6 days for SARS-CoV-2 vs 10.2 days for H7N9 (*P* = 0.86). One of the asymptomatic COVID-19 cases in our study, 59-year-old male patient, accompanying with hypertension and diabetes, was tested positive for both nasopharyngeal and stool specimens; while the duration of viral shedding in stool was up to 68 days.

## Discussion

The clinical presentation and laboratory indices at hospital admission were common in H7N9 and H1N1 patients, except that productive hemoptysis, thrombocytopenia were more common in H7N9 patients, those two factors have been associated with more severe outcomes [10]. Compared with features in patients with H7N9 and H1N1, patients with COVID-19 were more likely to exhibit the mild symptoms. Chinese Center for Disease Control and Prevention (China CDC) recently reported that most of the confirmed cases were classified as mild or moderate, 13.8% as severe, and only 4.7% as critically ill [11] Notably, the viral load that was detected in the asymptomatic patients was similar to that in the symptomatic patients [3] Recent research indicated that asymptomatic carriers can result in person-to-person transmission and should be considered a source of COVID-19 infection [12]. Unlike H7N9 and H1N1, the transmission of COVID-19 occurs during the prodromal period when those infected were mildly ill, and carry on usual activities, which contributes to the spread of infection.

The mean duration of viral shedding for nasopharyngeal specimens was 9.8 days for SARS-CoV-2 vs 9.9 days for H7N9, for severe cases, it was 10.6 days vs 10.2 days. It has been widely investigated that the duration of viral shedding in H1N1 ranged from 4 to 8 days [13–15]. It seems likely that SARS-CoV-2 infections were characterized by the prolonged viral shedding, compared with H1N1. SARS-CoV-2 viral RNA has been detected in the serum, urine, and feces of COVID-19 patients, but it is not known if this represents viral replication occurring outside of the respiratory tract [16]. Although the H7N9 patients had high mortality, there had been no confirmed cases of human-to-human transmission, and most infected humans had a history of contact with poultry or of having visited a wet market, and the outbreak of H7N9 had been controlled by integrative measures including the closedown of the wet markets in the affected areas [17–19]. However, the clinical spectrum of SARS-CoV-2 infection appears to be extensive, encompassing asymptomatic infection, mild upper respiratory symptoms, and spreading by respiratory and fecal–oral transmission.

According to the report of world health organization, the epidemic threshold of seasonal influenza was in December around the world, influenza activity decreased overall or returned to baseline levels in March for most temperate regions [20]. Worldwide, seasonal influenza A (H1N1) viruses accounted for the majority of detections. According to the report of China CDC, the mortality of influenza A (H1N1) diseases was less than 0.01% during the epidemic threshold period in china [21]. However, SARS-CoV-2 infections had a higher complication rate and more severe cases than H1N1 patients (Table 1). Furthermore, recent research reported that *R*_0_ of COVID-19 might be as high as 6.47 (95% *CI* 5.71–7.23) [22]. Likely, SARS-CoV-2 activity would not decrease with the change of seasons; long-term control measures are still needed.

Neutrophilia was more common in H7N9 and H1N1 patients, compared with COVID-19 cases. It maybe implied that patients with H7N9 and H1N1 were more likely to develop secondary bacterial infections. H7N9 cases had a higher proportion of glucocorticoids and antibiotics therapy. Increasing the risk of secondary infection, glucocorticoids could delay the clearance of coronavirus nucleic acids without lower hospitalization-fatality ratio for patients with H7N9 diseases [23]. Nevertheless, recent research suggested that timely and appropriate use of corticosteroids, together with ventilator support, should be considered for severe patients to prevent ARDS development [24]. According to the chest CT images of COVID-19 patients on admission, the mainly positive findings were various ground-glass densification shadows with multiple diffusions in both lungs, the pros and cons need to be weighed carefully before antibiotics and glucocorticoids treatment.

The mean time from illness onset to severity was 8.0 days for COVID-19 and 5.2 days for H7N9, it may imply that there was a therapeutic window that could be exploited, provided comprehensive treatment including an active antiviral agent was available. But, if not treated promptly, the asymptomatic or mild cases may develop severe pneumonia, even end up dead. The therapeutical emphasis of COVID-19 was to antiviral early that would decrease the peak viral load and delay the progression of lung lesions; thus this would reduce the hospitalization-fatality ratio [25].The mean time from illness onset to severity for H7N9 was shorter than COVID-19, the key to controlling disease progression was early detection and timely treatment.

Chronic heart disease more frequent in patients hospitalized with COVID-19, compared with H1N1 (Table 1), whereas for severe cases, COVID-19 had the highest prevalence of chronic heart disease than H7N9, which might be associated with increased secretion of ACE2 in the COVID-19 compared with H7N9 and H1N1. SARS-CoV-2 infection is triggered by binding of the spike protein of the virus to ACE2, which is highly expressed in the heart and lungs [26]. There have been hypothesized that the use of ACE-inhibitors and angiotensin receptor 1 blockers (ARBs) may have effect the course of COVID-19 [27, 28]. Nevertheless, further evidence are required before any recommendations are made about starting or withdrawing ACE-inhibitor and ARB medications.

The comparisons in this study are limited by a lack of parameters of the transmission dynamics. Further research is still needed if the epidemic features of COVID-19 are similar to Seasonal influenza A H1N1 or not.

## Conclusions

The proportion of severe cases were higher for H7N9 and SARS-CoV-2 infections, compared with H1N1.The meantime from illness onset to severity was shorter for H7N9, compared with COVID-19. This may imply that there was a therapeutic window that could be exploited before developing into severe cases. Besides, we found that chronic heart disease was associated with an increased risk of COVID-19 severe cases compared with H7N9 and H1N1. Also, the factor that the mild symptoms may contribute to the pandemic of COVID-19. Furthermore, according to the neutrophil responses and the typical CT findings, SARS-CoV-2 infections were less likely to develop secondary infections, which maybe suggest that the progression of COVID-19 is more insidious.

## Supplementary information

**Additional file 1: Table S1.** Logistic Regression analysis of Chronic heart disease

**Additional file 2: Table S2.** The number of lobes involved

**Additional file 3: Table S3.** The details of the virus shedding duration of nasopharyngeal swab for COVID-19 and H7N9

## Data Availability

The datasets used and/or analysed during the current study are available from the corresponding author on reasonable request.
