# Supplementary material for: Comparison of patients hospitalized with COVID-19, H7N9 and H1N1
Source: Infect Dis Poverty. 2020 Dec 2;9:163. doi: 10.1186/s40249-020-00781-5 (PMC7707904; doi:10.1186/s40249-020-00781-5)
Supplement: Supplementary file 1 — Additional file 1: Table S1. Logistic Regression analysis of Chronic heart disease [file 40249_2020_781_MOESM1_ESM.docx]

**Additional Table S1.** Logistic Regression analysis of Chronic heart disease

| Dependent variable | Control variable | Model χ² | P | OR（95%CI） |
| --- | --- | --- | --- | --- |
| COVID-19 vs H7N9 | Sex, Age, Smoking history, Hypertension, Diabetes | 27.693 | <0.001 | 10.08[2.15,47.34] |
| COVID-19 vs H1N1 | Sex, Age, Chronic renal disease | 16.045 | 0.003 | 5.13[1.44,18.26] |
| COVID-19 vs H7N9  (severe cases) | Sex, Age | 6.067 | 0.014 | 4.59[1.27,16.60] |
