# Supplementary material for: Comparison of patients hospitalized with COVID-19, H7N9 and H1N1
Source: Infect Dis Poverty. 2020 Dec 2;9:163. doi: 10.1186/s40249-020-00781-5 (PMC7707904; doi:10.1186/s40249-020-00781-5)
Supplement: Supplementary file 2 — Additional file 2: Table S2. The number of lobes involved [file 40249_2020_781_MOESM2_ESM.docx]

**Additional Table S2. The number of lobes involved**

| **The number of lobes involved** | **COVID-19**  **(*n* = 83)** | **H7N9**  **(*n* = 36)** | ***P* value*** | **H1N1**  **(*N* = 44)** | ***P* value^#^** |
| --- | --- | --- | --- | --- | --- |
| 5 | 21 (25%) | 20 (55%) | 0.01 | 4(9%) | < 0.01 |
| 3-4 | 30 (36%) | 8 (22%) | 0.14 | 4(9%) | < 0.01 |
| 0-2 | 32 (38%) | 8 (22%) | 0.08 | 36 (82%) | < 0.01 |

*P* value*: compared “COVID-19” and “H7N9”, P value**^#^**: compared “COVID-19” and “H1N1”.
